# An evaluation of two large scale demand side financing programs for maternal health in India: the MATIND study protocol

**DOI:** 10.1186/1471-2458-12-699

**Published:** 2012-08-27

**Authors:** Kristi Sidney, Ayesha de Costa, Vishal Diwan, Dileep V Mavalankar, Helen Smith

**Affiliations:** 1 Division of Global Health, Nobels Väg 9, Karolinska Insitutet, Stockholm, Sweden; 2 RD Gardi Medical College, Agar Road, Surasa, Ujjain, Madhya Pradesh, India; 3 Public Health Foundation of India– Gandhinagar (IIPH-G), Sardar Patel Institute Campus, Drive-in Road, Thaltej, Ahmedabad, Gujarat, India; 4 Liverpool School of Tropical Medicine (LSTM), Pembroke Place, Liverpool, United Kingdom

**Keywords:** India, Demand side financing, Maternal morality, Chiranjeevi yojana, Janani suraksha yojana

## Abstract

**Background:**

High maternal mortality in India is a serious public health challenge. Demand side financing interventions have emerged as a strategy to promote access to emergency obstetric care. Two such state run programs, Janani Suraksha Yojana (JSY)and Chiranjeevi Yojana (CY), were designed and implemented to reduce financial access barriers that preclude women from obtaining emergency obstetric care. JSY, a conditional cash transfer, awards money directly to a woman who delivers in a public health facility. This will be studied in Madhya Pradesh province. CY, a voucher based program, empanels private obstetricians in Gujarat province, who are reimbursed by the government to perform deliveries of socioeconomically disadvantaged women. The programs have been in operation for the last seven years.

**Methods/designs:**

The study outlined in this protocol will assess and compare the influence of the two programs on various aspects of maternal health care including trends in program uptake, institutional delivery rates, maternal and neonatal outcomes, quality of care, experiences of service providers and users, and cost effectiveness. The study will collect primary data using a combination of qualitative and quantitative methods, including facility level questionnaires, observations, a population based survey, in-depth interviews, and focus group discussions. Primary data will be collected in three districts of each province. The research will take place at three levels: the state health departments, obstetric facilities in the districts and among recently delivered mothers in the community.

**Discussion:**

The protocol is a comprehensive assessment of the performance and impact of the programs and an economic analysis. It will fill existing evidence gaps in the scientific literature including access and quality to services, utilization, coverage and impact. The implementation of the protocol will also generate evidence to facilitate decision making among policy makers and program managers who currently work with or are planning similar programs in different contexts.

## Background

The adoption of the Millennium Development Goals in 2001, specifically goal 5, renewed the global emphasis on reducing maternal mortality. This international commitment gained political attention; many low income countries prioritized improving women’s access to maternal health services [1].The global MMR decreased from 320 in 1990, and was 251 per 100 000 live births in 2008 [2]. Despite the international commitment, maternal mortality remains somewhat unchanged globally. In 2008, an estimated 342,900 women died world-wide from pregnancy related complications. More than half of these deaths occur in six low-income countries; one-quarter in India alone [2].

Between 1992 and 2006, the Indian Ministry of Health and Family Welfare focused on strengthening the health system infrastructure to better support emergency obstetric care (EmOC) services especially in the public sector. A concerted effort was made to increase capacity for institutional deliveries by upgrading facilities to improve access to skilled birth attendance and EmOC [3]. Key strategies included upgrading community health centers to function as first referral units, creating 24/7 access to health centers for delivery, and training and empowering non-specialist qualified medical doctors to administer anesthesia for emergency obstetric procedures [1]. In spite of this government investment, national surveys showed that the proportion of institutional deliveries only increased marginally from 26% to 39% during that period [4,5].

While it is extremely important to invest in EmOC facilities, developing strategies that increase the use of these services especially among the poor who suffer the largest burden of maternal deaths are equally significant [6]. Most deaths can be avoided by prompt access to EmOC services. However, despite strengthening of the supply side (facilities), poor women are still at risk as they face a number of barriers, particularly financial, to access EmOC in the absence of social safety nets and widely prevalent out-of-pocket payment mechanisms [7]. In 2005, the government recognized the need to prioritize maternal health amongst the poorest and set up the National Rural Health Mission (NRHM) specifically to improve access to care for low income people in rural areas.

Using the platform of the NRHM, the government introduced demand-side financing initiatives for maternal health to reduce financial barriers that often preclude women from accessing skilled attendance at birth [8]. Two large scale demand-side financing programs to promote institutional delivery, conditional cash transfers (CCT) and vouchers, are currently in operation. The intended outcome of both programs is to promote institutional delivery, thus reducing maternal mortality through improving access to EmOC.

A traditional CCT provides a monetary incentive directly to the intended group on the condition the beneficiary satisfies a pre-defined set of requirements [9]. India’s Janani Suraksha Yojana (JSY or Safe motherhood program), a nationwide program, is a conditional cash transfer to all women who deliver in public sector institutions. This program will be studied in Madhya Pradesh Province. Chiranjeevi Yojana (CY or Long life program) is operational only in the Gujarat province, and functions in the context of an existing strong private obstetric care sector. It is a voucher based system where private obstetricians are reimbursed by the government to perform deliveries of tribal women or those living below the poverty line. Vouchers, which tend to be cashless, are used to reduce the direct costs of healthcare and increase demand for services [10] (Table 1).

**Table 1:** Characteristics of two Indian demand-side financing programs for maternal health

	** *CY – a voucher program (Gujarat)* **	** *JSY – a conditional cash transfer (MP)* **
** *Area of Operation* **	Gujarat (60.4 million)	Nationwide (studied here in Madhya Pradesh) (72.6 million)
** *Context* **	Socioeconomically relatively advanced: MMR half of that in MP (142/100 000), 16% population below the poverty line	Poor socioeconomic indicators, MMR (310/100 000), largely rural province, 38% of the population below the poverty line
** *Target group* **	Mothers below poverty line and tribal mothers	All mothers
** *Type of Program* **	Payment by state **to the private provider i.e. obstetrician***(voucher based)*	CCT – payment by state to **the mother***(conditional cash transfer)*
** *Incentive for* **	Institutional delivery	Institutional delivery
** *Accepted place of delivery* **	Empanelled private sector obstetric facilities	Public sector institutions largely
** *Involvement of private sector* **	Yes	Yes (extremely restricted)
** *Payment mechanism* **	Providers paid per block of 100 deliveries (20% in advance)	Payment to woman at the time of discharge from hospital after delivery
** *Quantum of payment* **	A flat amount of INR 288000($5760) is paid to the obstetrician per 100 women (regardless of delivery type)	INR1400 ($28) to rural mothers on discharge (INR1000 to urban mothers).
** *Expected effect* **	Provides access to EmOC care – which is available more widely	Increase institutional delivery, hence access to EmOC
** *Support for Emergency transport* **	Centralized 108 ambulance system	Decentralized Janani Express model

This protocol describes the proposed comprehensive evaluation of two respective programs in Gujarat and Madhya Pradesh. The evaluation, called the MATIND project is a four year project supported under the EU FP7 framework. The proposal aims to study (i) trends in program uptake since inception(ii)the influence of the program on institutional delivery rates, maternal and neonatal outcomes, (iii) explore service providers’ experiences,(iv) emergency transportation systems for obstetric referrals,(v) characteristics of the facilities participating in the programs, (vi) quality of care administered, (vii)experiences of the beneficiaries and non-beneficiaries, (viii) the role of community health workers and (ix) the cost effectiveness of the programs.

The program evaluation will be conducted at three different levels of the health system with specific objectives at each level: (i) the provincial (policy) level which includes secondary data from governments and other important stakeholders at the provincial level, (ii) the district level (sub-provincial) which focuses on health facilities (iii) and the community level, in villages (sub-district), which includes mothers and local health workers.

The objectives at each level of the study are listed below and summarized in Table 2:

1. Provincial (policy) level:

(i) Program Trends: To describe trends in program uptake for each program since inception and to study district level time series of program uptake data from 2004 to 2011. Further to examine the extent to which changes in the proportion of institutional deliveries have influenced maternal and neonatal outcomes over that period of time.

(ii)Private Sector Participation: To describe trends in private sector (health facilities) participation in each of the programs over time. Also, to study the characteristics of private sector participant facilities.

With regard to the JSY program in MP, to explore the reasons for the decision to subsequently reduce the role of the private sector in the program. Also to explore among selected erstwhile participating private facilities, their experience with JSY and their perception on the state decision to exclude them from the scheme.

With regard to the CY program, to explore experiences and motivations among selected private providers currently participating in the program, reasons for non-participation among a sample of those who were eligible but never participated and those who participated initially but have since left the program.

(iii) Emergency transportation for obstetric care: To study two different emergency obstetric transportation models utilized in each of the programs with regards to coverage, equity in access, current utilization, and cost of the service. Further, to explore among a group of mothers their experience of gaining access to and using the transportation.

(iv) Cost-effectiveness of the Programs: To develop and apply a model for assessment of the utility of the two maternal health programs and to perform a sensitivity analysis by testing alternative assumptions for a cost utility analysis.

2. Facility Level (public and private facilities):

(v) Facility Survey: To list all facilities in the study districts offering obstetric care and classify them as non-EmOC, basic or comprehensive emergency obstetric care (BEmOC or CEmOC) facilities. Further, to study the characteristics of these facilities with regard to ownership, accessibility, bed strength, human resources, training, referral communications and processes, availability of equipment, drugs and supplies and infection control procedures.

(vi) Quality of Care: To study in EmOC facilities the quality of care through direct observation of normal deliveries, a review of case records and registers and assessments of provider competence. Further to study experiences and perceptions of quality of care of mothers who delivered under the respective programs, and to study key procedures they experienced. In cases of mothers who were referred into facilities, referral indications and transportation details will be studied.

3. Community level (mothers in sampled study villages):

(vii) Program beneficiaries and non-beneficiaries: To study and compare beneficiaries and eligible non-beneficiaries of the program in the community in terms of: socio-demographic characteristics, utilization of antenatal services, birth attendance, delivery outcome, birth weight, receipt of post natal care, immunization of the child and costs of care incurred. Reasons for participation/non participation and choice of place of delivery will be explored. Predictors of non-participation will also be studied.

(viii) Equity in access to facilities (geographic): To estimate the influence of distance to facilities on place of delivery in comparison to other determinants of delivery service use.

(ix) Role of community health workers in program uptake: To study the role played by the ASHA (accredited social health activist) and the traditional birth attendants (TBA) in the antenatal, delivery and postnatal periods of program mothers. Also to explore among ASHAs their motives for participation and long term professional aspirations within the NRHM. The dynamic between various community level workers, the TBA, the ASHA and the female health worker will be explored. Concerns on the potential duplication of roles between the latter two will be explored among policy makers.

**Table 2:** Summary of objectives and proposed methods at the implementation level

**Study level**	**Objectives**	**Proposed methods**
Provincial (Policy)	To study program utilization trends	Secondary data analysis - complete times series, Document reviews, Key person interviews with policy makers, program managers and private sector representatives, Stakeholder discussions
	To ascertain the influence of changes in ANC service utilization and institutional deliveries on maternal and neonatal outcomes	
	To study trends in private sector participation	
	Explore the motivations for participation/non participation with the private sector in CY program	
	To understand the exclusion of the private sector in the JSY program	
	Explore experience of private provider in JSY	
	Comparison of emergency care transportation systems	
	Cost effectiveness of the programs and the emergency transportation models	
District (Facility)	List and classify all facilities performing deliveries according to their EmOC functionality	Facility Survey, Observations of normal deliveries, Register and case record review, Case vignettes to study provider competence, In depth exit interviews with mothers
	Survey and describe facilities conducting deliveries	
	Assess the quality of care administered in study facilities	
	Study outcomes (type of delivery, maternal mortality and morbidity, foetal outcomes) among program beneficiaries and non-beneficiaries in study facilities	
	Experiences of public and private sector physicians of the programs (in terms of task load, shifting of tasks, human resources required, monetary transactions with the program)	
Community	Identify program beneficiaries and non-beneficiaries.	Questionnaires, In-depth interviews, Focus group discussion, Spatial methods (GIS) to study distance
	Study differences between background characteristics, geographic access to EmOC, outcomes (maternal), type of health service utilization in pregnancy (and delivery), use of emergency obstetric transportation services and expenditures during delivery	
	Identify predictors for program uptake	
	Compare infant health status and health service utilization between users and non-users.	
	Among users, study perceived quality of care at facility	
	Explore motives and barriers for participation/non-participation among eligible beneficiaries and non-beneficiaries	
	Understand the working dynamic for the community health workers(ASHA, TBA & female health workers)and their role in the program	

## Methods

### Setting

**Figure 1:**
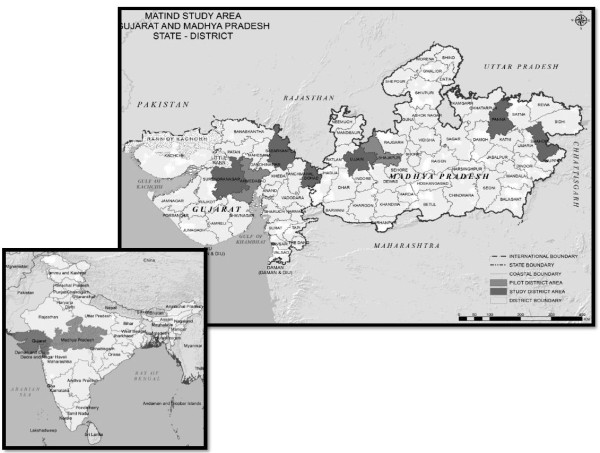
MATIND Study Area: Selected Study Districts in Madhya Pradesh and Gujarat.

The study will take place in two provinces (Figure 1) where the demand-side financing programs are operational, Madhya Pradesh and Gujarat. These provinces differ from one another both socio economically and with regard to other health related indicators (Table 3).

**Table 3:** Demographic and maternal health indicators of Gujarat, Madhya Pradesh

	**Population**	**Administrative districts**	**BPL**	**Literacy rate**	**Institutional delivery**	**IMR**	**MMR**
Madhya Pradesh	72.6[25]	50	38%[26]	71%[25]	47%[27]	62[28]	310[29]
Gujarat	60.4[30]	25	16%[31]	79%[32]	56%[33]	44[28]	142[28]

For studies at the district level, we purposefully selected three districts from each province in collaboration with the provincial ministries of health. The selected districts include districts from different geographic areas of each province, varying human development indices and represent different population sub groups including disadvantaged groups like scheduled castes (SC) and tribes (ST).

### Study design

A range of methods will be utilized during the data collection phase. Document reviews, time series, surveys, in-depth interviews, exit interviews, focus group discussions, case record reviews, and structured observations will be used. These are described in greater detail in the section below.

#### Data collection methods

1. Provincial (policy) level:

At this level we will collect secondary data on program related inputs, outputs and outcomes in each province. A time series analysis, consultations with key stakeholders, and review of relevant program documents will allow us to study program trends (objective i) and comment on the uptake of the programs since inception. A complete time series will be derived from maternal health data with regard to uptake (proportion of eligible women who participate), institutional delivery (total number of women delivering in a public or private facility), district level ante-natal care (ANC) coverage (proportion of women completing >3 ANC visits), and maternal and neonatal deaths. The data will be obtained from multiple sources including the District Level Household Survey (DLHS), Annual Health Survey, Sample Registration System (SRS), Vital Registration and health department sources for the purpose of data triangulation and the development of robust estimates. A major challenge in the analysis is the lack of complete time series for the maternal mortality data. Instead of relying on a single source for information, statistical data fusion techniques will be applied to collate data from various sources. The data fusion techniques will borrow strengths across time and space from the existing data to derive a complete time series estimates for the variables of interest.

A second study at the policy level will focus on trends in private sector participation (objective ii) in each of the programs over time. The role of private providers varies in each program. Private providers played a small part in the JSY program; however they are a large resource in Gujarat and thus the central focus in CY. Private facility characteristics will be studied through a cross-sectional facility review (see objective v). In MP, we will explore with NRHM program managers the experiences of collaborating with private providers and the perception of the role of the private sector in JSY, justification for criteria used for selection, and rationale for their inclusion in and subsequent exclusion from the program through in-depth interviews. Implementation issues and the overall benefits or disadvantages of participating in the program will also be explored with a selected group of private providers.

In Gujarat, we will use qualitative methods to explore participation of the for-profit health sector in CY and understand the factors influencing private provider participation. The qualitative research will be conducted using an interpretive approach, and will seek to understand private providers motives for participation/non participation, appropriateness of payment amounts under the program, procedures for reimbursement and interfacing with government officials. In addition, the study will explore how the program affects/affected patient turnover, what proportion of their patients are/were CY, influence on human resources, changes made to the facility to incorporate the program and suggestions on how to improve the functionality of the program. An experienced researcher will interview a purposive sample of private providers who (i) are currently participating in the program, (ii) were part of the program but no longer participate and (iii) never participated. Thematic analysis using the framework approach will be applied.

Difficulties accessing transportation to receive emergency obstetric care is one of the numerous barriers experienced by many rural women [11], and we plan to explore the emergency transportation alternatives for obstetric care (objective iii) in each of the programs using quantitative and qualitative methods. The two programs use different emergency transportation models both of which are public private partnerships initiated by their respective governments. The ‘108’ emergency transport scheme in Gujarat was launched in 2004. ‘108’ is a centralized, free, fully integrated emergency service ambulance that is equipped with trained staff and equipment to administer lifesaving care en route. It is a common transportation means used for all medical emergencies and disaster situations. Janani Express Yojana (JEY or Maternal Express Program), piloted in 2006 and scaled up in 2008, is utilized in Madhya Pradesh. It is also a free ambulance service; however it is used exclusively to transport obstetric cases to public facilities. It is a more basic form of transportation, and does not provide amenities to treat patients en route to the hospital. JEY is a decentralized service; vehicles are hired on a contractual basis locally at the district level and utilize a decentralized call center.

A quantitative retrospective data analysis will be completed with data ascertained from call centers to determine utilization patterns and uptake of the respective transport systems. Socio-demographic characteristics of the beneficiaries and other outcomes will be collected in a cross-sectional questionnaire to study equity in use of the service.

NRHM program officials and District Chief Medical & Health Officers (CMHOs) will be interviewed to gain insight into the structure under each model and the benefits and limitations of the respective decentralized and centralized emergency transportation services. The discussion will include perceptions on efficiency, geographical accessibility, functionality, sustainability, technical capacity, constraints and motivations of the human resources, and procedural limitations. In addition, costs associated with implementation and ongoing usage will be elicited.

All interviews will be conducted in Hindi by a research assistant, transcribed and then translated into English. In-depth interview data will be analyzed thematically, using the framework approach. The analysis will draw out differences between the two schemes in terms of implementation, stakeholder views of the schemes and experiences of utilization. A cost effectiveness analysis between the two programs is also planned.

A cost effectiveness of the two programs (objective iv) will be performed through two different methods. The first approach will be to compare the costs and outcomes of the two programs for matched groups of participants. The primary outcome will be the proportion of institutional delivery. Other outcomes to be analyzed will be the proportion of cesarean deliveries, antenatal check-ups and delivery outcomes (live births, stillbirths, miscarriages). The costs will primarily be from the payer perspective, the state, but also costs to the patients will be collected and analyzed. The second approach will be to analyze each system separately from an equity perspective, i.e. how costs and access to services are distributed according to categories of socio-economic status. In view of the complexity and multi-dimensionality of both the interventions and the outcomes, we will not aim to comprise the analysis in a single index number.

2. Facility Level:

At the facility level we will collect primary data on the types of facilities that are administering obstetric care under the programs (objective v), and the quality of care received (objective vi). These facilities will be visited to study maternal health outcomes. Both quantitative and qualitative methods will be utilized to achieve these objectives.

An initial list of all public and private facilities in the study districts will be obtained from government records. The list will be verified by site visits. Snowballing will then be utilized to identify any missing private facilities where obstetric care is provided. A modified Averting Maternal Death and Disability (AMDD) survey [12] will be administered to each facility by a member of the research team and the performance of EmOC functions will be ascertained by interviewing key staff. The facilities will be classified with reference to standards based on the UN and WHO definitions [13] as non-EmOC, Basic Emergency Obstetric Care (BEmOC) or Comprehensive Emergency Obstetric Care (CEmOC). The availability level of EmOC services will be ascertained with reference to the population; the number of CEmOC, BEmOC and non-EmOC facilities in the public and private sector and in the rural and urban areas will be calculated.

A cross-sectional survey will provide additional information on how the facilities function under the respective programs as well as aspects of quality of care provided. The assessment will include variables on infrastructure, accessibility, human resources, functional equipment, accessibility to drugs and supplies, referral communications and processes, infection control procedures and service statistics. This survey will provide the current operating state of the facilities. Descriptive statistics will be calculated for the areas mentioned above. These will be compared between public and private facilities and between the two programs.

Data on delivery outcomes for women admitted in the facility will also be collected over a five day period at each facility that had more than 10 deliveries in a month. A research assistant, assigned to the facility, will administer a short questionnaire to mothers who deliver within the defined time frame. Basic socio-demographic characteristics, pregnancy and delivery details, and indication for referral if appropriate will be elicited. More specific details of the same delivery will be obtained from the nurse in the labor ward. These will include type of delivery, presence of known pregnancy or delivery related complications, maternal and neonatal outcomes. This will provide information on the proportion of these events among program mothers. In the case of CY, it will allow a comparison with non-program mothers who deliver at the same time in the program hospitals. This study will provide a cross sectional snap shot of outcomes for program mothers. The proportion of program mothers who have complications, who undergo instrumental delivery, referred, who have adverse neonatal outcomes will be reported as will the number of maternal deaths during this period.

The Hulton et al. Quality of Care framework will be used to assess the quality of care (objective vi) from both the provision of care (facility perspective) and the experience of care (user perspective) [14]. Observations of normal deliveries, case record and register reviews, competency rating of service care provider, mother’s experience and the referral process will all be used to ascertain the quality of care being administered to obstetric patients.

Non-participant observation of normal deliveries will be performed on a sample of mothers in selected facilities. The observations will describe the technical care given during labor and delivery, infection control practices and the interaction between the staff and mother. A retrospective review of pre-defined indicators from a sample of case records will be performed in all study facilities to assess the quality of records maintained. The registers will also be assessed and the number of deliveries and procedures performed will be recorded. The competence of labor ward nurses providing obstetric care will be assessed utilizing case vignettes. The case vignettes will focus on normal labor and delivery. A short structured exit interview will be administered to all post-partum mothers enrolled in the study before discharge to determine which key procedures they underwent. The procedures included in the questionnaire will be based on common practices that may harm or benefit the mother during or after delivery, as described in the WHO evidence based practices [14,15]. A sub-set of women who participate in the exit interview will be selected for an in-depth interview that will take place in their homes within four weeks of delivery. The in-depth interview will explore her experience of delivery care under the program and explore potential aspects of care that may inhibit her utilization of the facility for a future delivery. Indications for referral and referral processes for women referred to facilities at different levels will also be studied. Mothers who have been referred will be interviewed to obtain details of indications for referral in, distance travelled, time taken, transport and costs.

For the observations, content analysis will be used to conceptualize and classify events, behaviors and actions to obtain a rich understanding of quality of care and explanation of how women experience care at facilities [16]. Basic descriptive statistics will be calculated for key indicators from the record and register review. With regard to provider competence, descriptive analysis will identify competence areas that require strengthening. The data will be analyzed using inferential statistics to compare scores between levels of facilities and qualification and experience of providers. The data from exit interviews will be analyzed to describe use of evidence based practices by level of facility. Qualitative data derived from the in-depth interviews with mothers will be analyzed using the thematic Framework approach [17]. The obstetric referrals, indications for referral and details of referral-transport, costs, distance, and time will be described by the level of facility using mean, standard deviation and tests of significance. The referral pathways of the maternal deaths will be mapped on GIS and buffer analyzed. The buffer analysis will be used to study referring facility locations with regard to transfer time to CEmOC facilities.

3. Community level (mothers in the study villages):

At the community level we will collect primary data from recently delivered mothers to compare outcomes between eligible beneficiaries and non-beneficiaries (objective vii) for each of the programs. A cross-sectional study will be conducted with women residing in the selected study villages who delivered in the last year. JSY program participation is defined as an institutional delivery in any public health facility. Program non participation is defined as a home or private facility delivery. CY program participation is defined as a below poverty line (BPL) or tribal mother who delivers in a CY approved facility. Program non participation is defined as BPL or tribal mother who delivers outside of a CY approved facility.

Stratified systematic random sample will be used to select the study villages in each district. The districts will first be stratified by blocks (administrative units) and then the villages in each block will be classified into two strata based on the size of the population. Villages will be randomly selected from each strata based on the population proportion. Given the different design of each program, the sample sizes will vary in each province.

There will be three points of data collection; (i) when the woman delivers, (ii) 15 days and (iii) 28 days post-delivery. The ASHA will administer a simple symptom based checklist to all mothers regardless of place of delivery to ascertain maternal morbidity, complications associated with the pregnancy and delivery, at the three time points. To ensure quality data collection, a physician will administer a second checklist to 5% of all mothers which will be compared to the data collected by the ASHA.

Socio-demographic details, history of previous pregnancies, receipt of comprehensive maternal health care (antenatal, intrapartum and post natal care), place of delivery, delivery type, birth weight, immunization of the child, indirect and direct costs associated with delivery, program participation and maternal and neonatal outcomes will be elicited post-delivery in the mother’s home by a trained research assistant. In addition, reasons for participation/non-participation in the program, reasons for their choice of facility and barriers (if any) to access of care will be collected. Data will also be collected on household amenities and assets. This will help create an indicator to assist in quantifying the degree of income related inequalities and how it influences the inequities in access to care. In the event of a maternal death, the head of the household will be requested to provide the information.

Propensity score matching will be used to select a comparison group from the sample of non-participants closest in terms of observable characteristics to a sample of program participants. Comparisons of the outcome variables described above between the two groups (users and non-users) will be studied. Reasons for participation/non participation will be described. In a separate hierarchical model, the predictors of non-participation will be studied in each program. Costs incurred will be compared by place of delivery.

The facilities identified in objective v will be mapped onto a Geographic Information System (GIS) (objective viii). Digitized GIS maps of the selected districts in both provinces will be created. The map will have basic layers of district and block boundaries, road and rail networks, and settlements. Global Positioning System (GPS) co-ordinates will be taken for all facilities and added to the map as a separate layer. The location of the mothers who participated or did not participate in the program (objective vii) will be added as an additional layer. Distance from the mothers’ village to the selected health center will be calculated from the GIS. The association between distance from a facility and program use will be studied. Level of care offered at the facility will also be used as a predictor in the model for choice of place of delivery. Suitable GIS software along with standard statistical software will be used. A regression model will be developed using the background predictors of place of delivery (maternal characteristics, socioeconomic situation) as well as distance to the nearest facility.

The last objective in this level will focus on the community health workers’ participation in the program (objective ix). The ASHA is a female resident of the village who is incentivized by the government to motivate women to deliver at facilities under the programs. The government has identified the ASHA as a pivotal role in the community success of the program [18]. A purposive sample of ASHAs and mothers from their villages will be identified and invited to participate in separate focus group discussions and individual in-depth interviews. The following areas will be explored within the two groups: their practical role in the program, experience recruiting and retaining mothers in the program (cooperation received and barriers encountered), influence on the decision making process of the mothers on where to deliver, types of services provided to the mother, the interaction with other ground level health workers, perception of how their activism is received within the community (voluntary, enforced or incentivized activism), and long term career aspirations. In addition, a sample of TBAs will be identified and interviewed about their role in the program and how they interact with the mothers, ASHAs and other community level health workers. Among NRHM program officials at state level, the risk and implications of overlapping roles in the community will be explored through in-depth interviews. Data from all interviews and group discussions will be analyzed using thematic Framework analysis [17].

#### Data management

##### Quantitative studies

Research Electronic Data Capture (REDCap) will be utilized as an online database. Data will be exported into appropriate statistical software for analysis. *Qualitative studies:* Transcripts and recorded material will be stored in a locked location. The interviewee will be disassociated from the transcripts before analysis.

### Discussion

Both of the proposed programs are innovative demand side financing programs with an explicit focus on promoting institutional delivery. They are the first of their kind implemented on such a large scale. Several demand-side financing interventions have been previously deployed to create demand for different health services [19]. Most of these schemes focus on vulnerable groups and have provided incentives for child education, immunization and nutrition. Demand side programs have varied from being small scale pilot programs to larger nationwide programs like the PROGRESA/Opportunities program in Mexico, the BolsaFamilia in Brazil and others in Latin America [20]. To date, there have been some government run national or sub national level demand side programs focused on maternal health, particularly in South Asia [21].

In this article we have outlined the protocol for an evaluation of the two large scale demand side financing programs for maternal health in India. There have been repeated calls in the scientific literature to study the effectiveness of such large demand side financing programs.

Design of the evaluation: Habicht et al [22] emphasizes the key influence of ‘why an evaluation is done’ on the subsequent design of the evaluation. The main reason for the proposed evaluation is to generate evidence on the performance and impact of these programs that is relevant to policy makers and program managers in India and other countries, who currently work with these programs or are considering initiating comparable programs in similar or different circumstances. From a country perspective, the evaluation will provide feedback on program performance and impact in the study provinces. It will also suggest areas that need to be strengthened. The evaluation aims to bridge evidence gaps in the scientific literature on the performance and impact of large scale demand side financing programs for maternal health.

The current evaluation studies both *performance and impact*[22] of the programs. This is a comprehensive design covering provision including access and quality to services, utilization, coverage and impact in comparison to reports on these programs thus far. A recent impact evaluation of the CCT program in India [23] based on secondary data reported a significant increase in institutional delivery. Previous literatures on the effects of CCT have demonstrated while the use of services show improvement, there is mixed evidence on the final outcome [20]. This evaluation also aims to study the influence of the programs on the maternal mortality outcome, which has not been studied before.

Multiple methodologies will be used in the evaluation to study the *performance* of the program. These will include quantitative analysis of secondary data, surveys of facilities, questionnaires to mothers, community based surveys of households, and spatial analyses to study access. Qualitative methods will also be employed. These will include in depth interviews with policy makers, program managers, healthcare providers and women. Focus groups with women who utilized the program will also be done. The project will contribute new knowledge in a number of areas which have not been studied before in the context of demand side financing programs; including spatial analysis to study access to EmOC, private sector collaboration in demand side programs, an assessment of potential models for emergency transport and quality of care under the programs.

The assessment of *impact* will be made from secondary and primary data. One measure of impact will be the influence of the programs on the final outcome i.e. maternal mortality. This will involve the construction of statistical estimates from secondary data sources as detailed on page 9. A second assessment of impact will be to study differences in outcomes among eligible beneficiaries and non-beneficiaries. While a randomized experimental study would have been the most robust design (balancing confounders between users and non-users), this is not feasible for these specific large programs as they have been rolled out nationwide/statewide. Hence, there are no non-program groups from which a random sample can be drawn. Given that the two programs are implemented completely across the provinces under study and so preclude a randomized design for evaluation, we will generate the comparison groups (counterfactual) using matched comparisons. In this evaluation, propensity score matching [24] will be used to select a comparison group from the sample of non-users closest in terms of observable characteristics to a sample of program participants.

In addition to performance and impact of the two programs, a *cost utility* analysis of each of the programs from a payer (state) and patient perspective will be done. There have been no previous economic analyses of these programs.

In summary, this paper describes a comprehensive evaluation protocol for two innovative demand side financing programs to promote maternal health in India. The protocol comprises an assessment of the performance and impact of the programs as well an economic analysis. The implementation of the protocol will generate evidence to facilitate decision making among policy makers and program managers who currently work with or are planning similar programs in different contexts. The evaluation also aims to fill existing evidence gaps in the scientific literature on the performance and impact of large scale demand side financing programs for maternal health. The results from the studies in this protocol will help support the design of efficient demand side financing programs in the future.

### Ethical approvals

The MATIND study has received ethical approval from institutional review boards at Karolinska Institutet, Liverpool School of Tropical Medicine, Public Health Foundation of India, and R.D. Gardi Medical College.

### Abbreviations

ASHA, Accredited Social Health Activists; ANC, Antenatal Care; ANM, Auxiliary Nurse Midwife; AMDD, Averting Maternal Death and Disability; BEmOC, Basic Emergency Obstetric Care; CY, Chiranjeevi Yojana; CEmOC, Comprehensive Emergency Obstetric Care; CCT, Conditional Cash Transfer; DHIS, District Health Information System; DLHS, District Level Household & facility Survey; EmOC, Emergency Obstetric Care; GIS, Geographic Information System; GPS, Global Positioning System; ICDS, Integrated Child Development Services; JSY, Janani Suraksha Yojana; MP, Madhya Pradesh; NRHM, National Rural Health Mission; OR, Odds Ratio; SC, Scheduled Caste; ST, Scheduled Tribe; TBA, Traditional Birth Attendant.

### Competing interests

The authors declare that they have no competing interests.

### Authors’ contribution

KS drafted the manuscript and all authors contributed subsequent drafts and revisions of the paper. ADC, DM, VD, RT, and HD were co-applicants on the original grant application and made substantial contributions to study design. The MATIND team worked with refining the protocol, development and testing of tools and the database. The MATIND study team includes: Lennart Bogg, Sarika Chaturvedi, Vinod Diwan, Hengjin Dong, Partha Ganguly, Veena Iyer, Kate Jehan, Rajesh Mehta, Rajesh Nair, Marie Ng, Santhi N.S., Parvathy Sankar Raman, Bharat Randive, Joanna Raven, Yogesh Sabde, and Rachel Tolhurst. All authors read and approved the final manuscript.

## Pre-publication history

The pre-publication history for this paper can be accessed here:

http://www.biomedcentral.com/1471-2458/12/699/prepub
